# Attachment disorder symptoms in foster children: development and associations with attachment security

**DOI:** 10.1186/s13034-023-00636-5

**Published:** 2023-08-11

**Authors:** Josephine D. Kliewer-Neumann, Janin Zimmermann, Ina Bovenschen, Sandra Gabler, Katrin Lang, Gottfried Spangler, Katja Nowacki

**Affiliations:** 1 University of Applied Sciences and Arts Dortmund, Dortmund, Germany; 2 University of Erlangen-Nuremberg, Erlangen, Germany; 3 Ruhr-University Bochum, Bochum, Germany; 4 German Youth Institute, Munich, Germany; 5 Child Guidance Center, Ingolstadt, Germany; 6 Ludwig Maximilian University Munich, Munich, Germany

**Keywords:** Reactive attachment disorder, Disinhibited social engagement disorder, Attachment, Foster care

## Abstract

**Background:**

Children in foster care constitute a risk population for developing symptoms of attachment disorders. However, little is known about the longitudinal course of attachment disorders and their association with attachment security in foster children.

**Method:**

This longitudinal study assessed attachment disorder symptoms in a sample of foster children (n = 55) aged 12 to 82 months. Foster parents with a newly placed foster child were assessed at three points during the first year of placement. At all assessment points, the Disturbance of Attachment Interview (DAI; Smyke and Zeanah in Disturbances of attachment interview, Tulane University, New Orleans, 1999) and the Attachment Q-sort (AQS; Waters and Deane in Monogr Soc Res Child Dev 50:41–65, 1985 German version as reported (Schölmerich and Leyendecker in Deutsche Übersetzung des attachment behavior Q-Set, revision 3.2. Unpublished manual, Ruhr University Bochum, Bochum, 1999) were used to investigate the interplay between disorder symptoms and attachment security.

**Results:**

The results revealed that the symptoms of attachment disorders decreased. The decrease was more pronounced for the inhibited than for the disinhibited symptoms with marked changes in the first 6 months of placement. There was a noticeable gender difference in the development with boys showing a more pronounced decrease in inhibited attachment disorder symptoms and a stronger increase of attachment security. After 12 months, no significant gender effects were found. Regarding the association between symptoms of attachment disorders and attachment security, a significant negative correlation between the inhibited attachment disorder symptoms and attachment security was found 12 months after placement.

**Conclusions:**

Attachment disorder symptoms decreased in the stable foster care environment. Thus, foster care seems to be an effective placement option regarding children’s attachment development.

## Background

Attachment disorder symptoms occur in pathological caregiving settings [34] and the developmental course responds to the caregiving environment [48, 30]. The current study examined the development of attachment disorder symptoms after placement into foster care, as well as the association with attachment security.

### Attachment disorders

DSM-IV distinguished a reactive attachment disorder with an inhibited and a disinhibited subtype. In general, the inhibited type is characterized by withdrawal, hypervigilance or ambivalent social behavior and an absence of attachment behaviors, whereas indiscriminate friendliness and contact seeking to unfamiliar adults are present in the disinhibited type [1]. Because of the differences in symptomatology and development, the two subtypes have been divided into two independent disorders in the DSM-5 [2]. The “Reactive Attachment Disorder of Infancy and Early Childhood” (RAD) refers to the inhibited type of attachment disorder, while the disinhibited type has been reframed under the concept of the “Disinhibited Social Engagement Disorder” (DSED).

### Attachment disorders and attachment security in foster care

RAD and DSED are rare disorders and have been found mainly in children who have experienced abuse or severe neglect/deprivation or had no consistent caregiver in their first months or years of life [4, 20]. Due to experiences of separation, abuse, and neglect, children in foster care constitute a risk population for developing symptoms of attachment disorders [49]. Placement in foster care serves to protect the children and is an important intervention of the German youth welfare support system with around 90,000 children p.a. growing up in this kind of placement in Germany [31]. Most studies addressing foster children’s psycho-social adjustment refer to foster children who have been living in their foster families for a longer time, while only few studies address the development in the first months of placement.

In the Bucharest Early Intervention Project (BEIP), inhibited symptoms decreased with a positive change of the caregiving environment [14], whereas the disinhibited symptoms decreased less quickly [30]. Further studies found persistence of disinhibited symptoms in children placed in foster or adoptive families [6, 15] and a stability of the pattern was assumed. In constrast, a more recent study indicates that inhibited as well as disinhibited symptoms tend to decrease after family placement [26]. Furthermore, in the BEIP, a reduction of DESD symptoms was associated with earlier placement in family care and less time in institutional settings [13].

In most studies investigating attachment security in foster children, the samples comprised children in their first year of life who had already spent some time in their foster families. In the meta-analysis by van den Dries, Juffer, van IJzendoorn and Bakermans-Kranenburg [35], foster children were found to be as securely attached as children living with their biological parents, but showed more disorganization than low-risk samples.

### Associations between attachment security and RAD or DSED

The inhibited attachment disorder symptoms have a phenotypical similarity to insecure, disorganized attachment [22]. Due to this similarity, van IJzendoorn and Bakermans-Kranenburg [37] highlighted the importance of attachment theory in understanding RAD. Some studies found no association between attachment security and both subtypes of attachment disorder symptoms [4, 23]. In constrast, Zeanah, Smyke, Koga and Carlson [44] found a negative correlation between attachment security, assessed in the strange situation procedure (SSP), and inhibited attachment disorder symptoms, but no significant association between attachment security and disinhibited symptoms [42, 43]. However, a recent meta-analysis examining the relationship between attachment and DSED symptoms [46] found that there is a small association between attachment insecurity and DSED symptoms.

### Aims of the present study

The field lacks studies investigating the longitudinal course of attachment disorder symptoms and their association with attachment security in foster children. Furthermore, most studies focused on foster children already living in their foster families for a longer time. The present study aims at investigating both attachment security and symptoms of RAD and DSED during the first year of placement in foster care. The first results reveal that the attachment security increased significantly within the first year of placement [17] leading to attachment security scores comparable to a normative sample of van IJzendoorn et al. [39] already six months after placement. In the present study, we address the interplay between both RAD and DSED symptoms and attachment security in the first year of placement. It has been shown repeatedly that inhibited symptoms tend to decrease after moving to a stable family placement [6, 14, 30]. Thus, it is assumed that inhibited symptoms decrease over time. For the disinhibited attachment disorder symptoms, it has been found that enhanced caregiving also correlated with a graduated decrease in symptoms [30]. Thus, it is expected that the disinhibited symptoms would decrease relatively slowly. Based on the finding of Zeanah and Gleason [42], a negative association between inhibited attachment disorder symptoms and attachment security was expected whereas it was expected that disinhibited symptoms of attachment disorder and attachment security are not correlated. Although gender differences have rarely been observed in attachment-focused studies, and -as far as we know- there are no published findings concerning attachment disorder symptoms, there are a few findings that indicate higher attachment security in girls compared to boys in high-risk samples [21, 24]. A pooled analysis by Gloger-Tippelt and Kappler [13] found gender to be a predictor for attachment representation with girls being more likely to be classified as securely attached. Thus, the association between gender and attachment remains interesting and therefore, the results will be tested for gender effects. It will be assessed whether gender interacts with the developmental course of attachment disorder symptoms or attachment security.

## Method

### Sample

The sample comprised 55 newly placed foster children with their primary caregivers during their first year of placement, which limited the possibility of participants to a smaller sample size. The main aim of the placement was to provide a long-term setting for children with difficult experiences in their families of origin. The participants were recruited through German social services departments around Dortmund, the Ruhr valley, and the Metropolitan region of Nuremberg. At wave 1, the children were aged between 12 and 82 months (M = 35.87; SD = 18.37) and 50.9% were female (n = 28). Before being placed with the current foster family, 85.5% of the children had lived in other foster families or institutions. Information on the foster children’s pre-placement experiences was obtained from the social workers of the social services department using a short self-designed questionnaire (see [17] for a precise description). The assessing questions were designed according to the Maltreatment Classification System [3]. In 83.6% of the cases (n = 46 emotional abuse had been a reason for the placement. Neglect was reported in 74.5% of the cases (n = 41. In most cases (n = 47,85.5% the foster mother was the primary caregiver.

### Procedure

The data presented in the current study was collected in a longitudinal design with three points of measurement (wave 1 to 3) throughout the first year of placement: on average 78 days (SD = 37.60) after placement into the foster family (wave 1), 6 months after placement (wave 2) and 12 months after placement (wave 3). Signed informed consent was obtained from all families preceding the first data assessment. At each wave, the caregiver-child dyads were observed twice within 2 weeks, once at home and once at the university. At home, the foster children and their foster caregivers were observed in a semi-structured videotaped 3-h visit. At the lab, structured observations were done and the *Disturbances of Attachment Interview* was conducted. All methods were assessed and coded by two assistants, with the second assistant being blind to earlier results or other measures.

### Measures

*Disturbance of Attachment Interview DAI; *[29]. The Disturbance of Attachment Interview is a semi-structured interview with the child’s primary caregiver. The manual contains 8 questions referring to the presence and extent of RAD and DSED symptoms. For each item, it is rated how strongly the described behavior is exhibited: clearly (score “0”), sometimes (“1”) or rarely (“2”). Signs of RAD were assessed with five questions with the overall score ranging from 0 to 10. Signs of DSED were measured with four items leading to an overall score ranging from 0 to 8. A high overall score thus indicates the presence of more symptoms. In this study, a German translation of the manual by Smyke and Zeanah [29, 27] was used. The structure of the factors of the translated manual was explored in a prior sample of foster children [16]. The transcripts of the interviews for the present sample were coded by three independent native-speakers. The coders were trained with a set of Portuguese cases translated into English from the lab headed by Isabel Soares, Portugal. The mean correlation with the Portuguese ratings was *r* = 0.80. Further, more difficult cases were discussed with the lab headed by Carlo Schuengel in the Netherlands and resolved by conference similar to Zeanah et al. [44]. Thirty percent of all interviews were coded by a second rater with a satisfying interrater-correlation of *r* = 0.76 for the inhibited scale and 0.80 for the disinhibited scale.

*Attachment Q-sort* (AQS; [40],German version [27]. The AQS assesses attachment in an extended observation in a home setting. It has been successfully applied in studies with infants as well as with children up to 88 months of age [7, 23]. The AQS provides continuous scores and is sensitive to changes over time. It may be used repeatedly with the same child and has been applied in groups of extremely disturbed children. In a meta-analysis, the validity of the AQS was proven [39].

After observing the child interacting with the current primary caregiver at home, two trained raters evaluated the behavior displayed by the child. 90 behavioral statements were evenly distributed on a nine-point Likert scale, whether they described the child exactly or not at all. The resulting profile of the child was correlated with an expert rating of a perfect securely attached child. The mean of the inter-rater reliability was 0.67 (SD = 0.11) at the first time of measurement, 0.67 (SD = 0.08) at the second and 0.69 (SD = 0.08) at the third.

## Results

### Descriptive values and intra-individual stability of attachment disorder symptoms and attachment security

*DAI*. The symptoms of RAD and DSED as measured with the DAI decreased within the first year of placement (see also [47]. The intra-individual stability for both disorders varied from moderate to high (see Table 1).

**Table 1:** Descriptive values and intra-individual stability of attachment disorder symptoms in the DAI

	DAI inhibited (0–10)											DAI disinhibited (0–8)									
	N	M	SD	*R*		Girls			Boys			M	SD	*R*		Girls			Boys		
				Wave 1	Wave 2	N	M	SD	N	M	SD			Wave 1	Wave 2	N	M	SD	N	M	SD
Wave 1	55	0.83	0.96			25	0.74	0.91	27	0.94	1.05	1.90	1.77			25	1.96	1.95	27	1.87	1.72
Wave 2	55	0.42	0.76	0.33*		25	0.72	0.98	27	0.19	0.40	1.27	1.45	0.60**		25	1.36	1.47	27	1.11	1.31
Wave 3	52	0.41	0.61	0.62**	0.54**	25	0.44	0.66	27	0.37	0.56	1.37	1.40	0.52**	0.42**	25	1.60	1.50	27	1.15	1.29

^**^p < 0.01, *p < 0.05

A repeated measure multivariate analysis of variance (MANOVA) with “wave” as the repeated measurement factor and “gender” as between subject factor was conducted. There was a main effect for “wave” revealing that the RAD symptoms significantly decreased over time, *F*(2, 100) = 9.06, *p* < 0.001, *ƞ*^*2*^ = 0.15. In addition, there was a significant interaction between gender and wave, *F*(2, 100) = 5.48, *p* < 0.01, *ƞ*^*2*^ = 0.10. The Least Significance Difference post-hoc tests (LSD) showed that the significant decrease occurred within the first 6 months of placement whereas no significant changes were found between waves 2 and 3. Because of the interaction effect of the factor gender, separate analyses were conducted for boys and girls. It was found, that in the subgroup of the girls, there was a more noticeable decrease from wave 2 to wave 3, while for boys, a significant decrease was found in the first 6 months of placement. For the boys, the symptoms had already diminished in the first six months to such an extent that no further decrease was subsequently observable.

Overall, the DSED symptoms declined significantly, F(2, 100) = 5.78, *p* < 0.01, *ƞ*^*2*^ = 0.10. The LSD post-hoc tests showed that the significant decrease occurred within the first 6 months of placement, whereas there was no significant change between waves 2 and 3. Regarding gender, there was no significant interaction effect: *F*(2, 100) = 0.37, ns., *ƞ*^*2*^ = 0.01.

*AQS.* The results on the development of attachment security are presented in Lang et al. [17]. There was a significant interaction between gender and wave for attachment security, *F*(2, 100) = 3.77, *p* < 0.05, *ƞ*^*2*^ = 0.07. At the first time of measurement, the attachment security scores of the girls were twice as high as the scores of the boys. The LSD post-hoc test showed that attachment security in boys increased from wave 1 to wave 2 and again from wave 2 to wave 3. After one year of placement, the boys were as securely attached as the girls (see Table 2).

**Table 2:** Descriptive values and intra-individual stability of attachment security in AQS (as reported for the total sample by Lang et al. [17]

	AQS security (−1 to 1)					Girls			Boys		
	N	M	SD	*R*		N	M	SD	N	M	SD
				Wave 1	Wave 2						
Wave 1	55	0.18	0.23			25	0.23	0.20	27	0.11	0.24
Wave 2	55	0.27	0.19	0.53**		25	0.31	0.18	27	0.22	0.19
Wave 3	52	0.31	0.21	0.32*	0.50**	25	0.30	0.21	27	0.33	0.21

^**^p < 0.01, *p < 0.05

### Association between DAI and AQS

Pearson's Correlation coefficients for the attachment security scores and RAD or DSED symptoms were calculated for all measurement points. At wave 1 and wave 2, there was no significant association between attachment security and symptoms of RAD or DSED. At wave 3, there was a significant negative correlation between attachment security and the RAD symptoms, *r* = −0.33, p < 0.05. At all three measurement points, DSED symptoms and attachment security were not significantly correlated.

## Discussion

Concordant with the hypothesis, the results show that symptoms of RAD and DSED decreased over time. Furthermore, the association between attachment security and inhibited attachment disorder symptoms changed over time. While there was no correlation immediately after placement, a negative correlation between attachment security and RAD was found one year placement. Attachment security and DSED symptoms were not significantly correlated. Gender effects were present in the developmental course of the inhibited symptoms.

### Describing the development of attachment disorder symptoms

Compared to Zeanah et al. [44], the mean scores of the foster children at wave 1 for both RAD and DSED fell between those of never institutionalized and institutionalized children. Compared to a sample of former institutionalized Romanian children now living in foster care by Gleason et al.[12], the present group of foster children exhibited fewer symptoms of RAD and DSED at the first time of measurement. Compared to a Dutch group of foster children, living in their foster families on average already for 3 years [23], the total number of foster children in the current study showing symptoms of RAD or DSED was twice as high at wave 1. However, the symptoms decreased significantly over time reaching a level comparable to non-clinical samples.

Considering the different types of attachment disorder symptoms, different patterns of development can be revealed: The disinhibited symptoms were more common at the time of placement and were more stable over time than the inhibited symptoms. The findings are congruent to what Rutter et al. [25] discussed concerning the finding that the inhibited symptoms are less common at the beginning of a foster family placement than the symptoms of DSED and seem to be influenced by experiences with the new caregiver more. The symptoms of RAD decreased in the foster environment and nearly reduced by half within the first 6 months of placement. Thus, similar to other studies [30, 48, 26], inhibited attachment disorder symptoms diminished quickly after placement in an adequate caregiving environment (see also [50] for a recent review).

However, also the DSED symptoms also decreased in the current sample. Other authors have found disinhibited symptoms such as indiscriminative behavior to be more persistent [6, 15, 28], but have also reported a positive influence of placement into a foster family compared to institutional care [13, 30].

### Interrelation between attachment security and RAD and DSED

In our data, no direct link was found between attachment security and DSED. The independence of attachment security scores and attachment disorder coding is supported by the findings of Oosterman and Schuengel [23] who used the same assessment methods.In addition, this finding is further evidence for the conceptual change in the DSM-5, in which DSED is no longer classified as an attachment disorder.

Regarding the RAD, there was a negative correlation with attachment security after one year of placement. This finding is in line with Zeanah et al. [44] who also found a negative correlation between inhibited attachment disorder symptoms and attachment security. In other studies, the foster family environment had a positive influence on attachment disorder symptoms [48, 30] and attachment security [19, 46]. The association seems to increase over time and a decrease in RAD symptoms is associated with an increase in attachment security. Therefore, the stable foster caregiving conditions reduced RAD symptoms and also supported the formation of a more secure attachment.

These results provide evidence for the distinction between the two former attachment reactive disorder types and are consistent with Zeanah and Gleason [43] who described RAD and DSED to be different regarding their association with attachment security.

### Gender effects

It was shown that the developmental course of the inhibited symptoms differed slightly by gender. It seems that the boys benefited more quickly from the positive changes in caregiving. One explanation might be that boys have a higher vulnerability during childhood [8, 41], but seem to benefit even more from a positive environment than girls, which was also found in an earlier study on German foster children [21]. This might be due to a higher susceptibility of boys concerning environmental factors. On the other hand, previous studies showed that indiscriminate behavior [18, 45], as well as other symptoms of RAD and DSED, as measured with the DAI [44], seem to be unrelated to gender. Thus, further studies are needed to shed light on the developmental pathways of attachment security and symptoms of RAD and DSED in both boys and girls.

Regarding attachment security there was a noticeable difference in the development of girls and boys. Over time, the boys caught up with the girls regarding attachment security and the gender differences vanished. The boys' attachment security seemed to be more effected by previous experiences but also profited more quickly from the stable caregiving environment in foster care. Gender differences in attachment behavior have rarely been observed in normative samples [36]. Only in a few studies including risk samples, girls showed higher security scores than boys on a representational level [21, 24].

Following the argumentation of Pierrehumbert et al. [24], one explanation for gender differences in attachment security might be that girls and boys show different social behavior when exposed to stress: Taylor et al. [33] proposed an alternative bio-behavioral response to stress than “fight or flight” for girls. To reduce vulnerability, the evolutionary evolved “tend-and-befriend” pattern involves caring for offspring and joining social groups in stressful situations. This behavioral pattern matches Bowlby’s [5] assumption, that stress exposure activates the attachment system and proposes closure between a child and her or his mother. McLaughlin et al. [19] found foster care to reduce symptoms of internalizing disorders in girls, but not in boys. Similar to the attachment behavior, also the assessment of inhibited symptoms might be influenced by girls tendency to “tend and befriend”. Thus, the pattern might not be seen so easily in girls.

Further studies investigating gender differences in the development of attachment disorder symptoms are needed.

### Limitations

The current study has several limitations: Firstly, the sample size is rather small and thus, the findings have to be interpreted with caution. Further, participation was voluntary and therefore, the results cannot be generalized for the whole population of foster children. Finally, although the longitudinal design provides the possibility to assess changes over time, potential learning effects due to repeated assessments cannot be excluded.

### Practical implications and future directions

Our findings reveal, that foster care in a family environment is able to increase attachment security and reduce attachment disorder symptoms. Thus, the family setting can improve children’s attachment development. In this sample, we found a small gender difference regarding the development of attachment disorder symptoms. The hypothetical higher susceptibility of boys concerning environmental factors and different stress responses for girls and boys might have influenced the developmental pathway of both attachment behavior and attachment disorder symptoms. In future studies, differences in attachment development and the development of attachment disorders between boys and girls should be considered, especially in high-risk groups. Furthermore, we would emphasize that further approaches should focus on the developmental pathways of boys and girls regarding attachment and attachment disorder symptoms. Especially for boys already the first month of placement into foster care seem to be extremely benefitting. Thus, future studies should address the early influences on attachment behavior just after placement and consider potential differences in the development for boys and girls.

Overall, the results support the idea of corrective attachment experiences. With regard to out-of-home care, foster family placement seems to provide health benefits.

Further research is necessary to define the specific conditions that supported the positive development in the majority of the foster children, further research is necessary. Due to the limitations of the present study, we also suggest further investigation of the developmental course of RAD and DSED and their associations with attachment security in foster children.

## Data Availability

The data-sets analyzed during this study are available from the corresponding author on reasonable request.
